# Locoregional treatment for colorectal liver metastases aiming for precision medicine

**DOI:** 10.1002/ags3.12689

**Published:** 2023-05-18

**Authors:** Harufumi Maki, Anish J. Jain, Antony Haddad, Mateo Lendoire, Yun Shin Chun, Jean‐Nicolas Vauthey

**Affiliations:** ^1^ Department of Surgical Oncology The University of Texas MD Anderson Cancer Center Houston Texas USA

**Keywords:** ablation techniques, circulating tumor DNA, colorectal neoplasms, hepatectomy, liver neoplasms, mutation, neoplasm metastasis

## Abstract

In patients with colorectal liver metastases (CLM), surgery is potentially curative. The use of novel surgical techniques and complementary percutaneous ablation allows for curative‐intent treatment even in marginally resectable cases. Resection is used as part of a multidisciplinary approach, which for nearly all patients will include perioperative chemotherapy. Small CLM can be treated with parenchymal‐sparing hepatectomy (PSH) and/or ablation. For small CLM, PSH results in better survival and higher rates of resectability of recurrent CLM than non‐PSH. For patients with extensive bilateral distribution of CLM, two‐stage hepatectomy or fast‐track two‐stage hepatectomy is effective. Our increasing knowledge of genetic alterations allows us to use them as prognostic factors alongside traditional risk factors (e.g. tumor diameter and tumor number) to select patients with CLM for resection and guide surveillance after resection. Alteration in RAS family genes (hereafter referred to as “*RAS* alteration”) is an important negative prognostic factor, as are alterations in the *TP53*, *SMAD4*, *FBXW7*, and *BRAF* genes. However, *APC* alteration appears to improve prognosis. *RAS* alteration, increased number and diameter of CLM, and primary lymph node metastasis are well‐known risk factors for recurrence after CLM resection. In patients free of recurrence 2 y after CLM resection, only *RAS* alteration is associated with recurrence. Thus, surveillance intensity can be stratified by *RAS* alteration status after 2 y. Novel diagnostic instruments and tools, such as circulating tumor DNA, may lead to further evolution of patient selection, prognostication, and treatment algorithms for CLM.

## Graphical abstract

This review article covers four topics that are important for surgeons to consider as they individualize multidisciplinary treatment for patients with colorectal liver metastases: parenchymal‐sparing hepatectomy and/or ablation; two‐stage hepatectomy; somatic gene alterations and canonical pathways; and posttreatment surveillance. The knowledge of tumor biology can alter treatment intensity, accurately predict patient prognosis, and help to determine whether extensive or repeated resection is justified. Also, the surveillance algorithm can be personalized according to tumor biology.

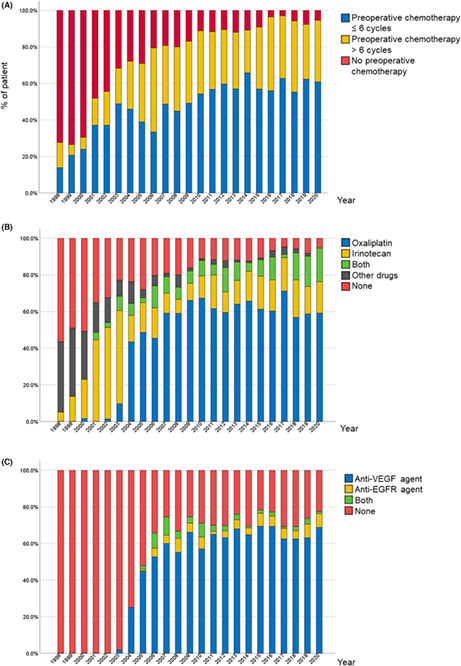

## 1 INTRODUCTION

In patients with colorectal liver metastases (CLM), surgery is potentially curative. The use of novel surgical techniques and complementary percutaneous ablation allows for curative‐intent treatment even in marginally resectable cases. The reported 5‐y overall survival (OS) rate after curative‐intent hepatectomy for CLM is ~50%.^1^, ^2^ Patients with CLM should be treated with a multidisciplinary approach, which for nearly all patients will include perioperative chemotherapy. Although optimal response to chemotherapy is significantly related to better survival, surgery for patients with suboptimal response is not a contraindication.

**FIGURE 1:**
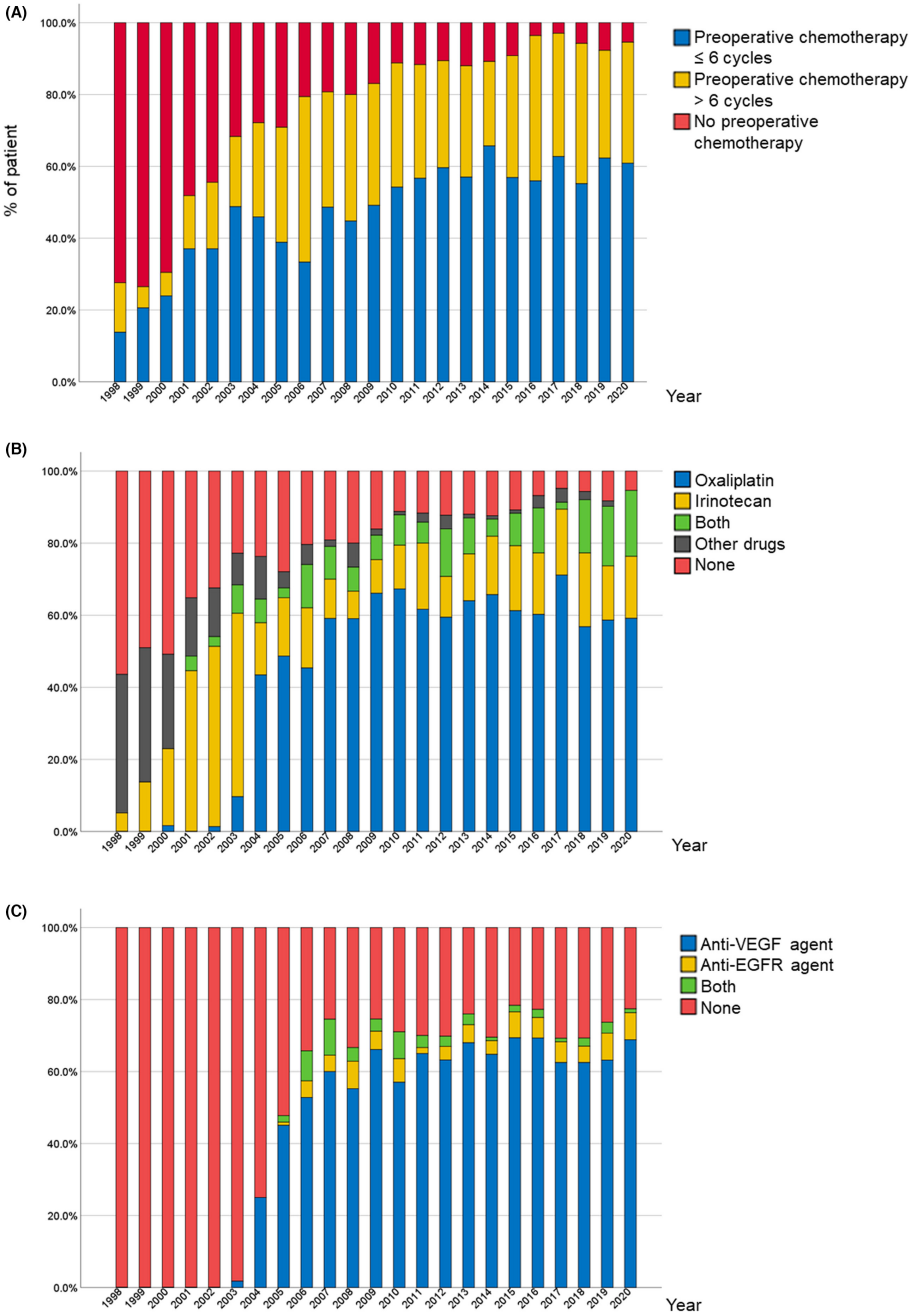
Chronological trends from 1998 through 2020 in preoperative chemotherapy for patients with colorectal liver metastases with respect to (A) number of cycles, (B) cytotoxic agents, and (C) molecular targeted therapy. VEGF, vascular endothelial growth factor; EGFR, epidermal growth factor receptor.

At the University of Texas MD Anderson Cancer Center, FOLFOX (leucovorin calcium, fluorouracil, and oxaliplatin) with bevacizumab for 8‐12 weeks and surgery 5‐6 weeks later is the standard preoperative treatment for the purpose of minimizing postoperative complications and maximizing treatment response according to the results of previous studies and the fact that bevacizumab is effective in all patients with CLM irrespective of *RAS* mutation status.^3^, ^4^, ^5^ In fact, preoperative chemotherapy was used in more than 80% of patients in 2007 and more than 90% of patients in 2020. In the majority of patients, the number of preoperative chemotherapy cycles was limited to six (Figure 1A). Since 2004, oxaliplatin‐based regimens have become the most frequently used regimens, given to almost 60% of patients who receive preoperative chemotherapy (Figure 1B). The vascular endothelial growth factor inhibitor bevacizumab is the first‐choice biological agent and has been administered to more than 60% of patients since 2011 (Figure 1C).

Patients with CLM are a heterogeneous cohort, and multidisciplinary treatment must be tailored for individual patients. The classic prognostic model used to select patients for CLM resection was developed in the late 1990s and included only clinicopathologic factors,^6^, ^7^ but somatic gene alterations have become increasingly used in the surgical decision‐making for patients with CLM.^1^, ^8^

In this review article, we discuss four topics that are fundamental in optimizing locoregional treatment of CLM: parenchymal‐sparing hepatectomy and/or ablation; two‐stage hepatectomy or fast‐track two‐stage hepatectomy; somatic gene alterations and canonical pathways associated with prognosis; and posttreatment surveillance.

## 2 PARENCHYMAL‐SPARING HEPATECTOMY AND/OR ABLATION FOR SMALL CLM

Considering recurrences after resection for initial CLM occur in ~70% of patients,^9^ it is important to develop a treatment strategy that anticipates repeat locoregional treatment for recurrent CLM. Therefore, the need for parenchymal‐sparing hepatectomy (PSH) and/or ablation is increasing, especially for small CLMs distant from the hilar plate.

### 2.1 Parenchymal‐sparing hepatectomy

PSH is recommended rather than non‐PSH because PSH has been shown to result in better survival and higher rates of resectability of recurrent CLM.^10^ Some studies have reported improved outcomes of non‐PSH versus PSH^11^, ^12^; however, these studies were not performed using cohorts appropriately matched by number or size of CLM.

**FIGURE 2:**
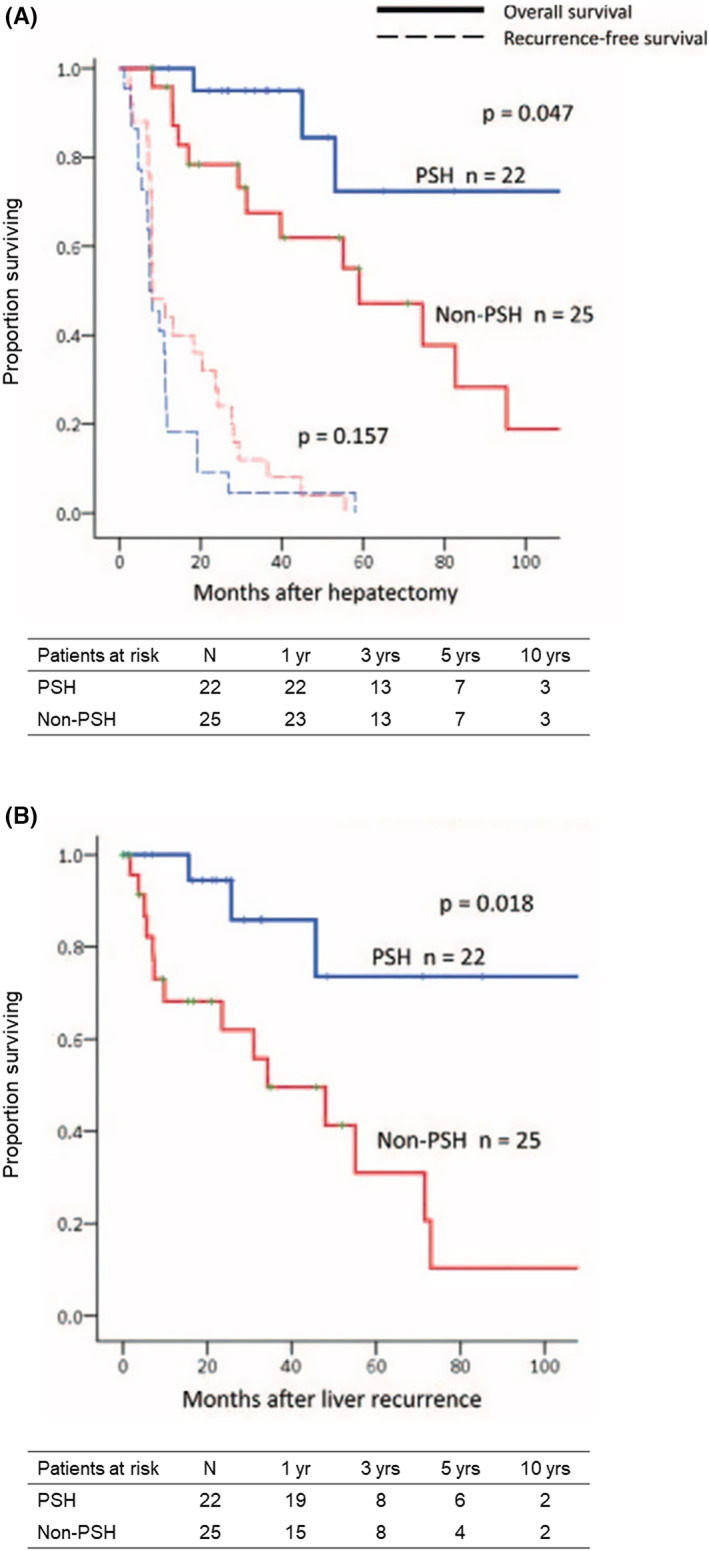
Survival in patients with liver‐only recurrence after parenchymal‐sparing hepatectomy (PSH) and non‐PSH for colorectal liver metastases (CLM). (A) Overall survival and recurrence‐free survival after initial hepatectomy. (B) Overall survival after the diagnosis of liver recurrence. (Adapted from Mise et al^13^ with permission.)

Mise et al^13^ compared PSH with non‐PSH in patients with solitary CLM measuring less than 30 mm and found that patients who underwent PSH had better survival from the date of operation and from the date of recurrence compared to patients who underwent non‐PSH (Figure 2). The reason was that repeat hepatectomy for recurrent CLM was more frequently performed in the PSH group than in the non‐PSH group (in 68% vs 24% of patients with recurrent CLM, *P <* 0.01). That is, PSH maintained “salvageability.”

In patients with small CLM, preoperative chemotherapy can render the CLM invisible on cross‐sectional imaging and make it difficult to identify the CLM intraoperatively. Thus, at MD Anderson Cancer Center, for CLM less than 2 cm in diameter that are not located on the liver surface, placement of fiducial markers is recommended before preoperative chemotherapy. Passot et al^14^ reported on 41 CLM in 32 patients who underwent percutaneous fiducial placement followed by resection or ablation. Nineteen of the 41 CLM (46%) were not visible on cross‐sectional imaging, but all 41 CLM were resected or ablated, and no recurrences were noted after median follow‐up of 14 mos.

Nishioka et al reported that in patients with CLM who underwent R0‐intent resection, the rate of local recurrence (recurrence at the resection margin) was not related to either surgical margin width or somatic gene mutation status, and that OS was not related to surgical margin status.^15^ The study implied that surgical margin width should not be tailored according to tumor biology to reduce the rate of local recurrence.

### 2.2 Ablation

At MD Anderson Cancer Center, ablation is guided by cross‐sectional imaging because it allows for optimal identification of tumor extent and close monitoring of ablation margins. In 2016, Shady et al^16^ reported that ablation improved local tumor progression–free survival in patients with ablation margins wider than 5 mm. In 2017, Odisio et al at MD Anderson Cancer Center reported that minimum ablation margins narrower than 5 mm and alteration in an *RAS* family gene (hereafter referred to as “*RAS* alteration”) were independent predictors of worse local tumor progression–free survival (PFS) (hazard ratio [HR] 2.48, 95% confidence interval [CI] 1.31–4.72, *P =* 0.006, and HR 3.01, 95% CI: 1.60–5.77, *P =* 0.001, respectively).^17^ Therefore, as local tumor PFS is conditioned by margins and *RAS* mutational status, 5‐mm margins after ablation will be suitable for patients with wildtype *RAS*, but patients with mutant *RAS* or unknown *RAS* status will need margins of at least 10 mm to reduce the risk of local recurrence. In a subsequent retrospective study at MD Anderson that analyzed outcomes when ablation margins were monitored using state‐of‐the‐art 3D imaging, *RAS* alteration was no longer an independent risk factor for local recurrence,^18^ suggesting that the ablation margin may have been appropriately performed using state of the art 3D reconstruction.

### 2.3 Planned incomplete resection with completion ablation

MD Anderson Cancer Center now uses a sequential treatment strategy for patients with extensive distribution of CLM, consisting of a planned incomplete (R2) resection followed by postoperative image‐guided percutaneous completion ablation of the remaining, intentionally untreated lesions.^19^ In a retrospective study comparing outcomes between this completion ablation strategy and standard intraoperative ablation, Okuno et al found that the 5‐y cumulative incidence of local tumor progression was significantly lower in the completion ablation group than in the standard intraoperative ablation group (31.7% vs 62.4%, *P =* 0.030), whereas the 5‐y OS rate did not differ between groups (53% for completion ablation vs 42% for intraoperative ablation, *P =* 0.41). The complication rate was also significantly lower for completion ablation (31.7% for completion ablation vs 62.4% for intraoperative ablation, *P =* 0.03).^20^ The study suggested that postoperative ablation can avoid the risk of resection for small CLM that are difficult to approach intraoperatively.

## 3 TWO‐STAGE HEPATECTOMY FOR BILATERAL CLM

Bilaterally distributed CLM pose special challenges. In 2000, Adam et al proposed treating such CLM with “two‐stage hepatectomy” (TSH) to achieve R0 resection and avoid postoperative liver failure due to a small future liver remnant.^21^, ^22^ In the typical TSH, the first‐stage operation involves minor resections of metastatic lesions within the left liver. This is followed by embolization of the right portal vein. After regeneration and adequate hypertrophy of the left side of the liver, a formal right hepatectomy is performed as the second‐stage operation.

At MD Anderson Cancer Center, 148 of 1779 patients (8.3%) with preoperative oxaliplatin‐ and/or irinotecan‐based chemotherapy for initial CLM underwent both stages of TSH during 1998–2020 (Table 1). Among them, 111 patients (75.0%) underwent portal vein embolization. Chemotherapy was not routinely administered during the interval between the first and second stages because it can prolong recovery after the first stage. Of note, postoperative chemotherapy was typically administered because of their extensive disease. Median (interquartile range) survival after completion of TSH (*n =* 148) was 4.2 (2.2–13.1) y, and the 3‐ and 5‐y OS rates were 60.2% and 45.5%, respectively.

**TABLE 1:** Steps in multimodality therapy in patients treated perioperatively with oxaliplatin‐ and/or irinotecan‐based chemotherapy and hepatectomy at MD Anderson Cancer Center, 1998–2020 (*n =* 1779)

Treatment step					Number of patients
1	2	3	4	5	
CTX	HEP	CTX			1498
CTX	PVE	HEP	CTX		133
CTX	HEP	HEP	CTX		37
CTX	HEP	PVE	HEP	CTX	111

Abbreviations: CTX, chemotherapy; HEP, hepatectomy; PVE, portal vein embolization.

In a separate study, our group found that in patients who required a major hepatectomy, hepatectomy combined with ablation was associated with a lower 5‐y OS rate than TSH (24% vs 35%, *P =* 0.01), a higher rate of postoperative major morbidity (32% vs 14%, *P =* 0.003), and a higher incidence of postoperative hepatic insufficiency (28% vs 6%, *P <* 0.0001). This study shows that the use of simultaneous ablation should be avoided in patients undergoing major hepatectomy.^23^

One of the major disadvantages of TSH is dropout prior to the second‐stage hepatectomy because of the long interval between stages. To shorten the interval, we set up a hybrid room that combines the capabilities of a standard operating room with those of an interventional radiology suite.^24^ This room contains a fluoroscopy table and incorporates both a robotic C‐arm computed tomography (CT) system and a multislice CT scanner. The hybrid room enables us to perform the first‐stage hepatectomy, portal vein embolization, and CT imaging during one operation.

With the use of the hybrid operating and interventional radiology suite, the second operation can be performed within 4 weeks after the first operation.^24^ This innovative and accelerated approach is referred to as “fast‐track TSH.” Nishioka et al from our institution reported preliminary results of a cohort of patients who underwent fast‐track TSH and found that there were no deaths within 90 d after operation, the median kinetic growth rate was 2.9% per week, and the median interval between stages was 5.6 weeks.^25^ Our results compared favorably with the historically high mortality rates reported with associating liver partition and portal vein ligation for staged hepatectomy (ALPPS).^26^ Thus, ALPPS has not been performed in any of the more than 3000 patients who have undergone hepatectomy for CLM at MD Anderson.

## 4 GENE ALTERATIONS ASSOCIATED WITH PROGNOSIS AFTER CLM RESECTION OR ABLATION

Somatic alterations are important for guiding treatment for patients with CLM. Somatic alteration status can guide decision‐making regarding ablation margins; the appropriateness of resection at the initial presentation of CLM and at recurrence; and the frequency of postoperative surveillance (Table 2). Chun et al^27^ reported that *TP53* was the most frequently mutated gene in CLM, mutated in 65.6% of patients, followed by *KRAS* (48.1%), *APC* (47.4%), *PIK3CA* (15.0%), and *SMAD4* (11.7%).

**TABLE 2:** Decision‐making for colorectal liver metastasis based on the evaluation of somatic gene alterations

	Clinical scenario	Decision based on somatic gene alteration
1	Treatment based intensity	Ablation margin
2	Treatment at initial presentation	Extensive resection versus chemotherapy
3	Treatment at recurrence	Resection versus chemotherapy
4	Surveillance	Postoperative follow‐up imaging and treatment

**FIGURE 3:**
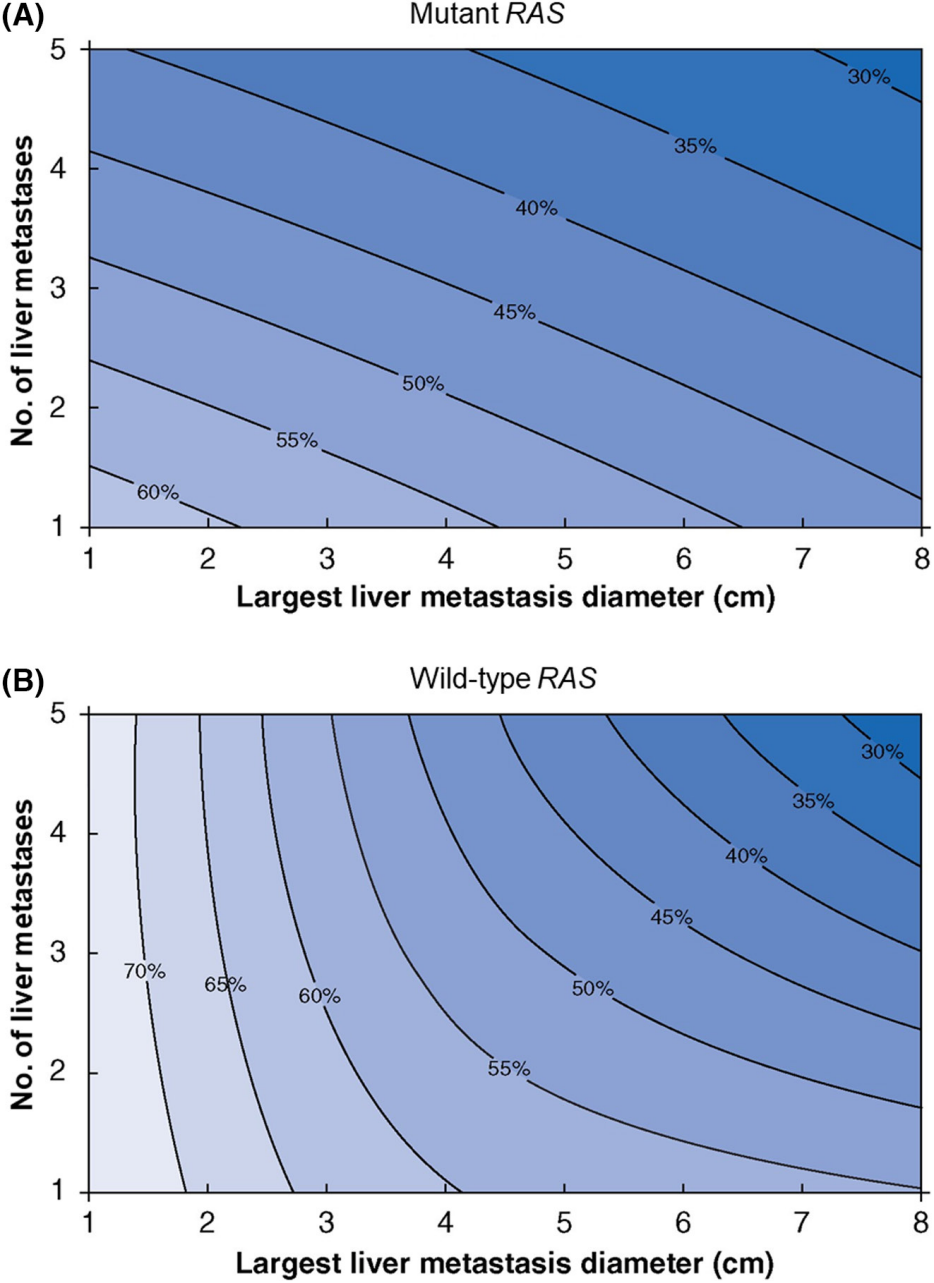
Contour plot of 5‐y overall survival probability according to the largest diameter and number of colorectal liver metastases for patients with: (A) mutant *RAS* and (B) wildtype *RAS*. (Adapted from Kawaguchi et al^1^ with permission.)

Patients with *RAS* alteration have worse survival than those with wildtype *RAS* after curative‐intent hepatectomy. In 2021, Kawaguchi et al^1^ created a contour prognostic model for survival after CLM resection using data from the MD Anderson cohort (*n =* 810) (Figure 3), similar to the “metroticket” model for survival after liver transplant in patients with hepatocellular carcinoma based on tumor number and size.^28^ The Kawaguchi et al model was validated in an international multicenter cohort, and these results illustrated the negative impact of *RAS* alteration on outcomes after resection of CLM. Additionally, in an analysis of patients with planned TSH, 81.7% of whom had completion of TSH, Passot et al found that the median OS was significantly longer in patients with wildtype *RAS* than in those with mutated *RAS* (8.5 vs 2.8 y, *P <* 0.001).^29^

In addition to *RAS* alteration, *TP53* alteration and *SMAD4* alteration were reported to be independently associated with worse prognosis.^8^, ^30^ Kawaguchi et al found that if patients had a *RAS* alteration but no *TP53* or *SMAD4* alterations, their prognosis was similar to that of patients with wildtype *RAS* after adjustment for *BRAF* mutation status, size of CLM, and surgical margin status (HR: 0.95, 95% CI: 0.55–1.65, *P =* 0.858).^8^ In terms of rarer gene alterations, *BRAF*‐V600E alteration, but not *BRAF*‐non‐V600E alteration, was associated with worse prognosis.^31^ Kawaguchi et al reported that *FBXW7* alteration was detected in 5.7% of patients and was associated with worse OS (HR: 1.99, 95% CI: 1.15–3.45, *P =* 0.015).^32^ On the other hand, *APC* alteration was associated with better survival.^33^, ^34^

**FIGURE 4:**
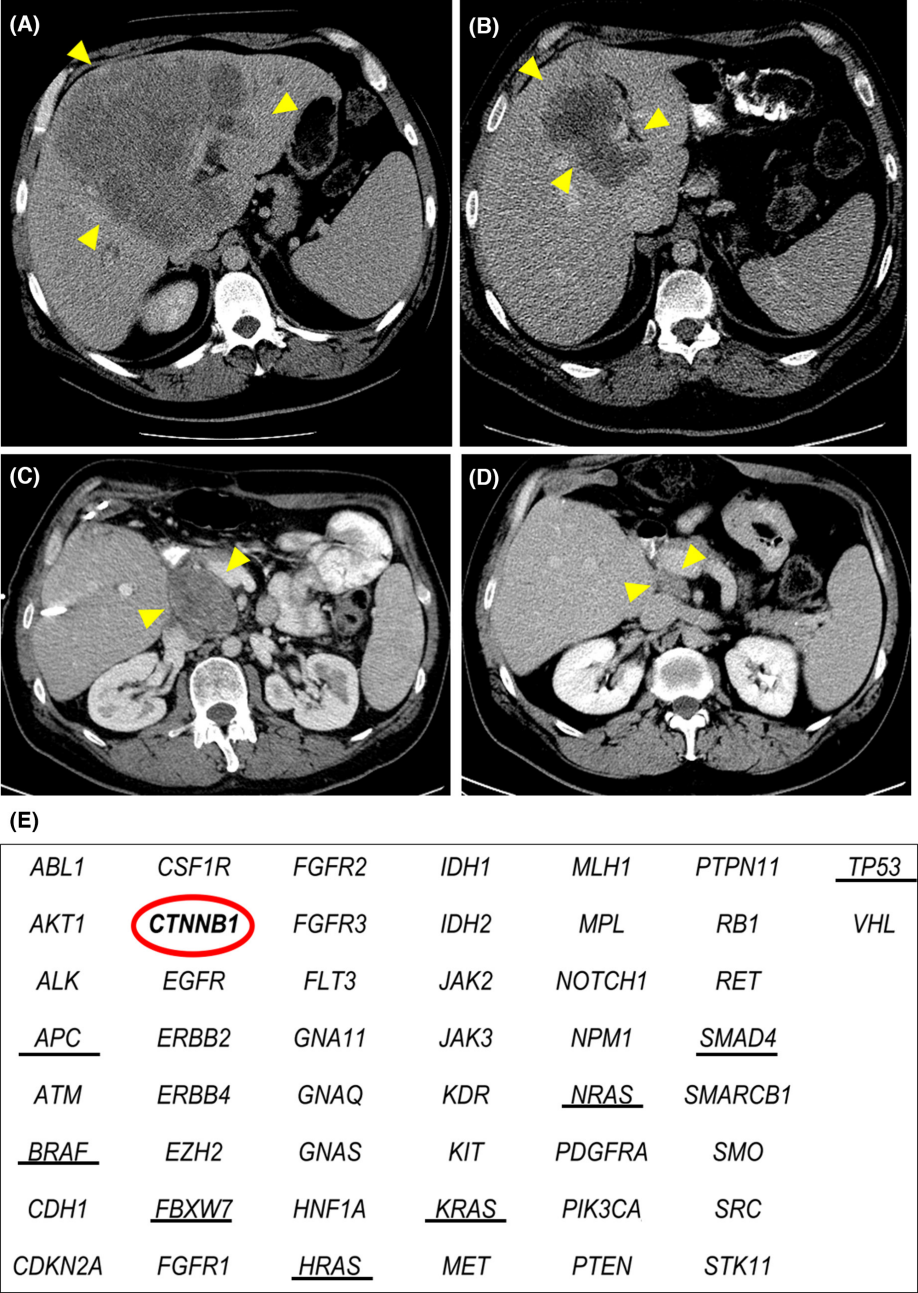
Disease course in a patient who presented with colorectal liver metastases (CLM) centrally located across both hepatic lobes and the caudate lobe after previous resection of primary colon cancer. Yellow arrowheads indicate the tumor. (A) Computed tomography (CT) image at the time of the initial visit showing a large tumor centrally located extending to the hilar plate and caudate lobe. (B) CT image after preoperative chemotherapy showing partial response with persistent invasion of the hilar plate and caudate lobe. (C) CT image 7 mo after extended left hepatectomy with common bile duct and caudate lobe resection showing recurrence in a retro‐portal lymph node. (D) CT image after chemotherapy showing partial response. (E) Results of gene panel analysis of 50 genes. The red circle indicates *CTNNB1* alteration of the tumor, and black underlines indicate driver genes associated with oncologic outcome after resection of CLM. Please note the absence of driver gene alteration in keeping with the good prognosis observed in this patient.

Analysis of gene alterations may be useful in determining whether an extensive operation to resect CLM is warranted. In patients with gene alterations associated with worse postresection survival, systemic chemotherapy might be more appropriate, whereas in patients without such alterations, aggressive surgical treatment can result in long‐term survival. The following case illustrates this principle. A 42‐y‐old man who had previously undergone resection of primary colon cancer, T3N1 according to pathologic staging, presented with CLM centrally located across both hepatic lobes and the caudate lobe (Figure 4A). After 10 cycles of therapy with FOLFOX and bevacizumab, a partial response was obtained (Figure 4B). Extended left hepatectomy with common bile duct and the caudate lobe resection was performed because disease invaded the hilar plate. Seven mo later, a recurrence was identified in a retroportal lymph node (Figure 4C). After 12 cycles of therapy with XELOX (capecitabine and oxaliplatin), a partial response was obtained again (Figure 4D). Lymphadenectomy was performed because the recurrent lesion was localized. At the most recent follow‐up, 7 y after lymphadenectomy, there had been no evidence of recurrence even though the patient did not receive chemotherapy. Gene panel analysis covering 50 genes revealed a *CTNNB1* alteration, but no *RAS*, *TP53*, or *SMAD4* alterations (Figure 4E). The prognostic impact of *CTNNB1* alteration has not been elucidated in CLM. This case suggests that genetic mutation analysis may be useful in determining whether an extensive, highly invasive operation is warranted.

**FIGURE 5:**
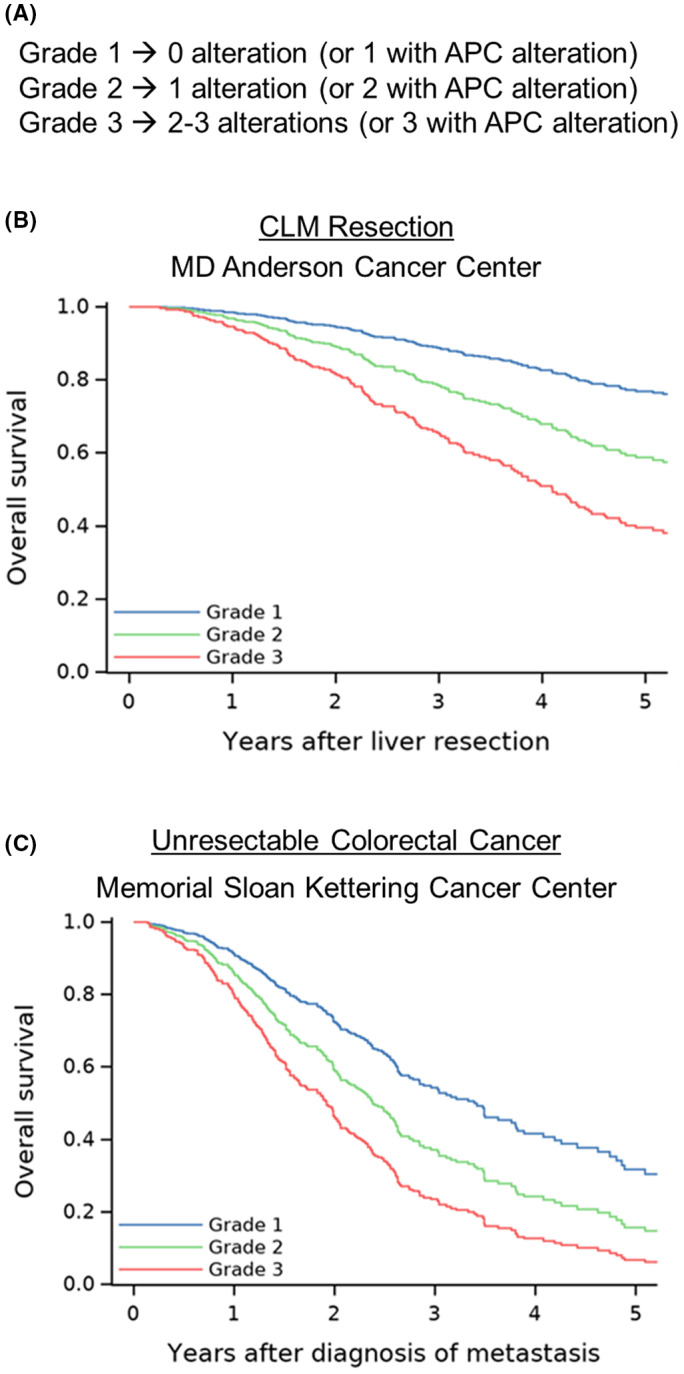
Pathway‐centric risk classification for patients with colorectal liver metastases. (A) Grades assigned according to *APC*, *TP53*, *RAS/BRAF*, and *SMAD4* mutation status. (B) Overall survival by grade. (C) Overall survival by grade in a validation cohort from Memorial Sloan Kettering Cancer Center. (Adapted from Kawaguchi et al^33^ with permission.)

Somatic gene alterations can be categorized into 10 canonical pathways: cell cycle, Hippo, Myc, Notch, NRF2, phoshatidylinositol‐3‐Kinase/Akt, receptor tyrosine kinase (RTK)‐RAS, transforming growth factor beta (TGFβ) signaling, P53, and β‐catenin/WNT.^35^ On the basis of this stratification, Kawaguchi et al reported that alterations in four pathways, p53, RTK‐RAS, TGFβ, and Notch, and their corresponding predominant genes (*TP53*, *RAS/BRAF*, *SMAD4*, and *FBXW7*) were significantly associated with worse OS after CLM resection, while alterations in the predominant gene of the β‐catenin/Wnt pathway, *APC*, were associated with better OS after resection of CLM.^33^ With these findings, the authors developed a pathway‐centric risk classification with three grades (Figure 5A) and demonstrated that higher grade was associated with significantly worse 5‐y OS (76.9% for grade 1 vs 58.7% for grade 2 vs 39.5% for grade 3) (Figure 5B). This model was validated in an external cohort of patients with unresectable CLM from the Memorial Sloan Kettering Cancer Center (Figure 5C).

In the context of ablation, Paolucci et al^36^ reported that alterations in the TGFβ pathway were associated with increased risk of development of new intrahepatic tumors (HR: 2.75, 95% CI: 1.39–5.45, *P =* 0.004) after initial ablation, and alterations in the Wnt signaling pathway increased the odds of salvage locoregional therapy at the time of intrahepatic progression (HR: 5.8, 95% CI: 1.94–19.5, *P =* 0.003).

## 5 POSTTREATMENT SURVEILLANCE

The principal aim of surveillance after resection of CLM is to permit early detection of disease recurrence and thus enable physicians to deliver repeated locoregional therapy in a timely manner. In patients with recurrence in the liver or lung after CLM resection, repeat hepatic or lung metastasectomy along with chemotherapy for recurrence is associated with better survival than chemotherapy alone.^37^, ^38^ However, frequent follow‐up tests and imaging are associated with increased medical costs. Therefore, the surveillance protocol should be based on the patient's risk of recurrence.

According to the National Comprehensive Cancer Network's 2020 guidelines, the follow‐up interval after resection for stage IV colorectal cancer can be extended starting 2 y after resection because ~70% of recurrences occur within the first 2 y.^9^, ^39^, ^40^ For instance, the guidelines recommend that oncologists perform serum carcinoembryonic antigen measurements and axial imaging every 3–6 mo until 2 y after resection and every 6–12 mo starting 2 y after resection. The risk factors associated with recurrence within the initial 2 y after resection of CLM include primary lymph node metastasis, greater number and size of CLM, and *RAS* alteration. However, the only risk factor associated with recurrence beyond 2 y after resection is *RAS* alteration.^41^ The data suggests that in patients with *RAS* alteration, the more frequent surveillance should be maintained beyond the initial 2 y after hepatic resection because of the increased risk of recurrence.

Circulating tumor DNA (ctDNA) may be useful for surveillance after CLM resection. ctDNA refers to small fragments of DNA shed by cancer cells into the bloodstream. These fragments can be detected in a patient's blood sample and may provide information about the presence, type, and progression of cancer. Fluctuations in serum levels of ctDNA can be used to detect minimal residual disease and monitor cancer progression, thus allowing for improved selection of treatment, prediction of treatment response, and detection of recurrence. For stage II or III colorectal cancer, a large prospective trial has demonstrated that adjuvant chemotherapy can be omitted in patients with confirmed negative ctDNA postoperatively.^42^

**FIGURE 6:**
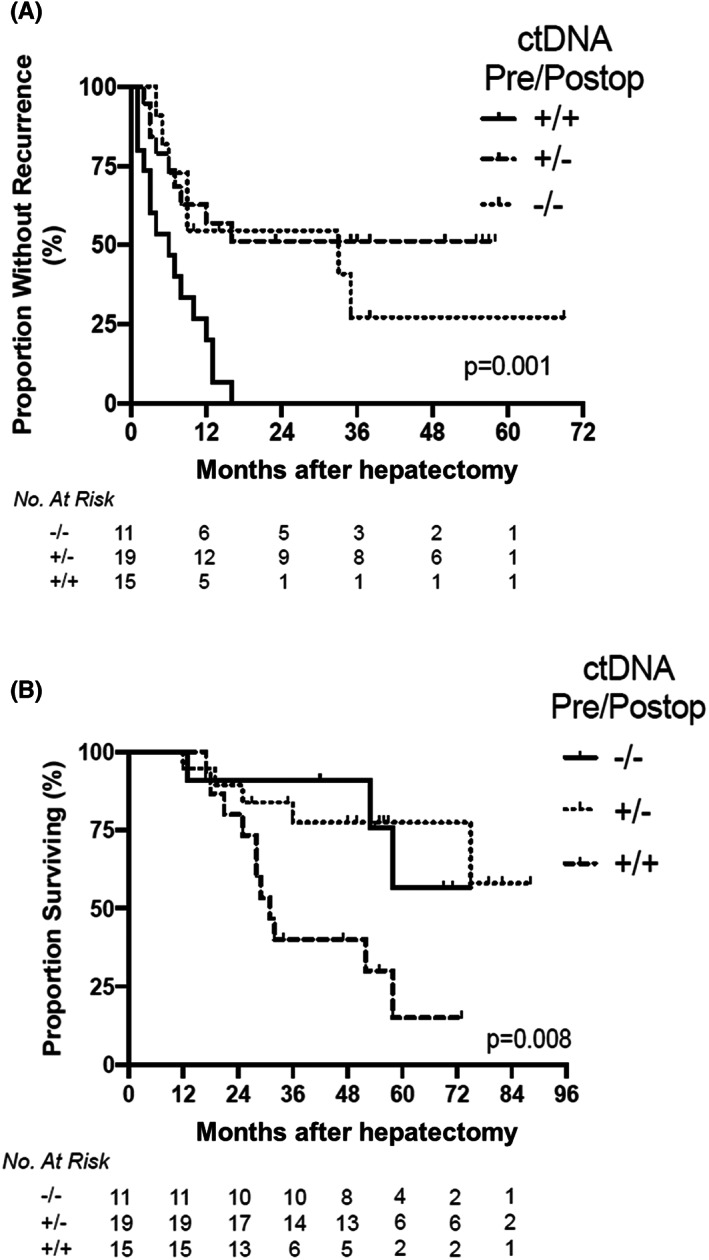
Recurrence‐free survival (A) and overall survival (B) by perioperative ctDNA dynamics among patients who underwent curative‐intent surgical resection of metastatic colorectal cancer including hepatectomy. Log‐rank *P*‐values. Pre/Postop, preoperative/postoperative. (Adapted from Newhook et al^43^ with permission.)

For CLM, Newhook et al performed a prospective study of changes in ctDNA over time in patients with CLM and found improved survival in patients who were negative for ctDNA before and after surgery. Patients who were positive for ctDNA before surgery but negative for ctDNA after surgery had survival similar to that of patients who were negative for ctDNA both before and after surgery (Figure 6A,B).^43^ Similarly, Øgaard et al^44^ reported that patients positive for ctDNA after surgery had a lower recurrence‐free survival than patients negative for ctDNA. They also reported that ctDNA status was more useful to predict recurrence than the serum carcinoembryonic antigen level. Moreover, recurrence was detected earlier by ctDNA than CT scan. That is, 24% of patients had inconclusive findings on CT scans during the follow‐up period, and ctDNA status at the time of inconclusive CT scans predicted recurrence.^44^ At MD Anderson, a phase II clinical trial (NCT05062317) is evaluating postoperative chemotherapy intensity and recurrence‐free survival at 12 months stratified by the status of postoperative ctDNA. The aim of the study is to determine if ctDNA‐negative patients can avoid continued intensive postoperative chemotherapy and its adverse effects.^45^

## 6 CONCLUSION

This article covered four topics that are important for surgeons to consider as they individualize multidisciplinary treatment for patients with CLM: parenchymal‐sparing hepatectomy and/or ablation; two‐stage hepatectomy or fast‐track two‐stage hepatectomy; somatic gene alterations and canonical pathways associated with prognosis; and posttreatment surveillance. The knowledge of tumor biology can alter treatment intensity, accurately predict patient prognosis, and help to determine whether extensive or repeated resection is justified. Also, the surveillance algorithm can be personalized according to tumor biology.

## AUTHOR CONTRIBUTIONS

HM, AJJ, AH, ML, YSC, and J‐NV: Study concept and design, article review, data analysis and interpretation, article preparation, article editing, final approval.

## FUNDING INFORMATION

Supported by the National Cancer Institute under award number P30CA016672, which supports the MD Anderson Cancer Center Clinical Trials Support Resource.

## CONFLICT OF INTEREST STATEMENT

The authors declare no conflicts of interest for this article.

## ETHICAL APPROVAL

This study was performed in accordance with the Declaration of Helsinki.
